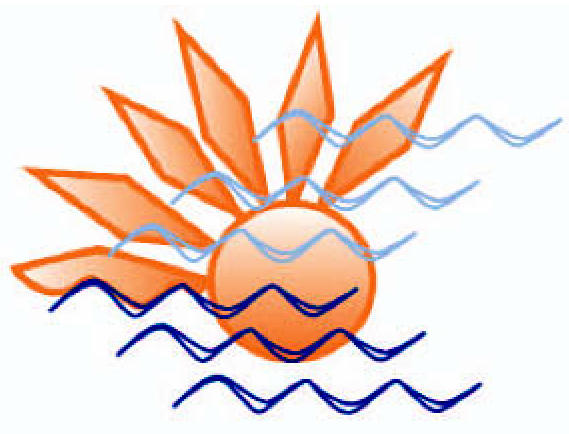# EHPnet: Climate Institute

**Published:** 2007-04

**Authors:** Erin E. Dooley

The Washington, DC–based Climate Institute is a nonprofit organization whose mission is to inform a broad spectrum of decision makers around the world about climate change, to raise international awareness of the issue, and to identify practical means of achieving significant reductions of greenhouse gas emissions. Toward these ends, the organization sponsors conferences, symposia, and ministerial briefings. As the Climate Institute has evolved, it has also begun promoting clean and renewable energy sources as a way to reduce greenhouse gas emissions. These efforts are described online at **http://www.climate.org/**.

The homepage is divided into several sections, including Steps Forward and Steps Back. These two sections contain information ranging from commentaries to meeting reports that describe achievements and challenges in the quest to lessen adverse human influence on the climate. There is also a What’s at Risk? section that addresses issues including how climate change is already affecting the Arctic and how it is likely to affect storm, flood, and drought activity in North America. Popular Culture is the focus of yet another section of the site featuring information on films about climate change as well as articles on topics such as how religious groups are becoming involved in the climate change issue and how climate change has become a popular theme even for cartoons.

The Programs section of the site describes the Climate Institute’s work in climate impacts, energy, international cooperation on climate change issues, and “environmental refugees”—people displaced as a result of problems such as drought, erosion, desertification, and deforestation. The institute also sponsors the Gordon MacDonald Environmental Leadership Program, which offers topical seminars and helps place students and recent graduates in internships and research positions. If monies are secured, the program will also fund the salary of a scientist or policy maker to work at the Climate Institute as well as the travel and living expenses of young scientists from participating institutions as they work on projects with the potential for a significant public impact.

Links in the left-hand column include tools for individuals, such as a personal environmental impact calculator that estimates the yearly impact of individuals and families in the areas of transportation, recycling, water usage, and energy usage. Topic links for sea level rise, extreme weather, ecosystems, air quality, ozone depletion, human health, and more lead to essays and other resources on how these topics relate to climate change. The site also offers educational materials for students from kindergarten through postdoc level. Resources are grouped by age, with an additional section on useful graphics and other teaching tools such as a climate impacts map.

## Figures and Tables

**Figure:**